# Beliefs about Polypharmacy among Home-Dwelling Older Adults Living with Multiple Chronic Conditions, Informal Caregivers and Healthcare Professionals: A Qualitative Study

**DOI:** 10.3390/healthcare9091204

**Published:** 2021-09-13

**Authors:** Marion Bieri, María del Río Carral, Marie Santiago-Delefosse, Giorgia Miano, Fanny Rosset, Henk Verloo, Filipa Pereira

**Affiliations:** 1 School of Health Sciences, HES-SO Valais/Wallis, 5 Chemin de l’Agasse, CH-1950 Sion, Switzerland; henk.verloo@hevs.ch (H.V.); filipa.pereira@hevs.ch (F.P.); 2 Research Center for the Psychology of Health, Aging and Sport Examination, Institute of Psychology, University of Lausanne, Quartier Mouline, CH-1015 Lausanne, Switzerland; maria.delriocarral@unil.ch (M.d.R.C.); marie.santiago@unil.ch (M.S.-D.); 3 Institute of Psychology, University of Lausanne, Quartier Mouline, CH-1015 Lausanne, Switzerland; giorgia.miano@unil.ch (G.M.); fanny.rosset@unil.ch (F.R.); 4 Service of Old Age Psychiatry, Lausanne University Hospital, 21 Rue du Bugnon, CH-1011 Lausanne, Switzerland; 5 Institute of Biomedical Sciences Abel Salazar, University of Porto, 4050-313 Porto, Portugal

**Keywords:** older adults, informal caregivers, home-dwelling, polypharmacy, management, daily medication practices, lived experience, beliefs, qualitative study

## Abstract

Although home-dwelling older adults are frequently assisted with polypharmacy management by their informal caregivers, they can still face medication-related problems. Identifying older adults’ and their informal caregivers’ beliefs about medication is a gateway to understanding and improving medication adherence. This study aimed to analyse beliefs about polypharmacy among home-dwelling older adults with multiple chronic conditions and their informal caregivers, focusing on their daily medication practices. Semi-structured interviews were conducted with 28 older adults, 17 informal caregivers, but also 13 healthcare professionals. Based on an inductive methodological approach, data were analysed using thematic content analysis. Interviews revealed the different attitudes adopted by older adults and their informal caregivers in relation to the treatment information provided by healthcare professionals. A variety of beliefs were identified and linked to medication adherence by examining daily medication practices. Polypharmacy was experienced as a habit but also an obligation, highlighting some of the strategies and negotiations underlying medication use at home. Collecting viewpoints from multiple stakeholders is an innovative way of accessing and analysing beliefs about polypharmacy. Daily medication practices provided information about medication beliefs and may contribute to developing targeted professional interventions that improve medication adherence.

## 1. Introduction

Between 2019 and 2050, the proportion of people in Switzerland aged 65 years old or more should rise from 18.7% to 25.6% [1]. Although many lead active lifestyles, they also ever more frequently live with chronic illnesses and comorbidities [2,3,4], and these may imply multiple complex medication regimens [5]. Polymedicated older adults (OAs) face a greater risk of medication-related problems (MRPs) [6,7] such as inappropriate prescriptions, drug–drug reactions, medication side-effects, incorrect administration or non-adherence to treatment [8]. Informal caregivers (ICs) often help home-dwelling OAs to manage their chronic polypharmacy, thus becoming true partners with home-care medical teams [9,10,11].

Managing chronic polypharmacy in the home could almost be considered an intrinsic part of the Instrumental Activities of Daily Living (IADL) [12,13,14]. That medication practices can be integrated into specific spatiotemporal contexts shows just how quotidian they are in nature and how they can transform a home space into a care space [15]. Beyond the apparent normality of living with polypharmacy, overlapping positive and negative attitudes about it underline the existence of ambivalent beliefs: OAs recognise medication as useful, helping to ensure their better health, but at the same time, some negative beliefs persist and lead to fears about drug–drug interactions, side-effects and medication overconsumption [12,16,17,18]. The chronicity often inherent in polypharmacy can create feelings of frustration: prescriptions can be open-ended or long-term, even when OAs’ health statuses may temporarily no longer require them [16]. Nevertheless, this continuity over time is a source of empowerment and tends to build up OAs’ sense of responsibility and self-discipline [19]. The diverse attitudes and beliefs regarding chronic medication help make up the fragile equilibrium that coexists with feelings of duty and an acceptance of the way things are [16,20].

Personal beliefs are at the origin of some patients’ personal preferences or wishes when it comes to medication treatments. Patient preferences are generally considered when prescriptions are modified, an approach that fits in well with the rationales of patient empowerment and shared decision-making processes [21,22,23]. This stance is supported by the Montreal Model and refers to the participation between carers and the patient discussing different options concerning the patient’s health, most notably risks and the patient’s values and preferences [24,25]. There is currently a consensus that patients are generally favourable to having some of their medication deprescribed [26,27], especially when they suffer from multi-morbidity and are polymedicated [28,29]. Using so-called implicit approaches, where the emphasis is put on the patient’s own judgement and opinions rather than on predefined criteria about their prescription, healthcare professionals (HPs) question the appropriateness of medication by looking at the individual patient rather than at their medication and illness [30]. Shared decision-making involves considering the positions adopted by the carer and the patient, with the latter choosing to participate more or less actively or passively. OAs position themselves differently along this participation continuum: they may be quite satisfied with simply being informed about decisions regarding their prescriptions, or they may prefer to be fully involved in participating in decision making [31]. Even though most OAs seem to want to participate actively in prescription decision-making processes, they often adopt a more backseat role once they have to manage more than four medical conditions simultaneously [32]. This positioning suggests either greater dependence on the prescriber in this complicated context or less confidence in their personal capacities for dealing with complex shared decision-making processes.

Although managing polypharmacy among OAs is now the subject of scientific research, there remain numerous gaps in the literature. Polymedicated OAs’ treatment adhesion has been well studied because of the increased risks of poorly following the instructions on prescriptions, especially in terms of dosage amounts and timings [33,34]. Nevertheless, few studies have examined beliefs about polypharmacy at home from the starting point of patients’ daily lived experiences [16]. Yet beliefs linked to medication treatments are a gateway to understanding the patient rationales underpinning their daily medication use [20]. Furthermore, most scientific studies have concentrated on specific illnesses and medications in order to explore particular non-adherent behaviours [17] and patient preferences [35]. Yet experiences of medication practices and the beliefs that surround them seem to go beyond specific types of medication for chronic illnesses [17,36]. ICs beliefs about polypharmacy have rarely been examined despite their essential role in the polypharmacy management of home-dwelling OAs [10,37]. It thus seems imperative to collect the viewpoints and beliefs of OAs and their ICs so as to include both groups in future public health recommendations, especially with the onrushing demographic and epidemiological transition currently facing Western countries. Discharge home after a period of hospitalisation is associated with changes in OAs’ medication regimens and disruptions to their regular daily routines [15,38]. This is why discharge home provides a privileged moment for exploring their beliefs about their medication consumption and the meanings associated with those beliefs.

The present article’s objective is to explore and analyse polymedicated home-dwelling OAs’ personal beliefs about and stances on their medication prescriptions. We do this from the starting position of their daily medication practices and the perceptions of the HPs who look after them. Although numerous socio-cognitive models in the field of health psychology are centred around the notion of beliefs, this term has rarely been adequately defined [39]. Within the framework of this study, we adopted a socio-constructivist perspective of the psychology of health so that we could define the notion of belief as “*a structure of socialized feeling, contingently allied to discursive practices and positions*” [39] (p. 945). According to this approach, beliefs can be examined in relation to the affective, bodily and socio-cultural dimensions of individuals’ lived experiences. Using this contextualisation enables us to understand OAs’ beliefs in relation to how they feel physically and affectively without decontextualising their subjective stances as social actors.

## 2. Materials and Methods

### 2.1. Study Design

The present study explores the issue at hand from a socio-constructivist perspective [40], according to which individuals’ life experiences can only be understood through their social interactions. It uses a qualitative methodology to examine individuals’ language as the preferred means of accessing their experiences, emotions and social interactions. Semi-structured individual interviews were conducted with OAs and their HPs, and semi-structured joint interviews were conducted with them and their ICs, when available. The present study is rooted in an interdisciplinary approach mixing health psychology and the nursing sciences, and it forms a part of a larger research project whose protocol has been published elsewhere [41]. The present qualitative study followed the COnsolidated criteria for REporting Qualitative research (COREQ) [42]. The completed 32-item COREQ checklist is annexed (Supplementary File S3).

### 2.2. Participants

The study used a purposive sampling approach and was conducted in the canton of Valais in French-speaking Switzerland. With the help of the local hospital centre and the community healthcare centre, the research team recruited 28 polymedicated home-dwelling OAs, recently hospitalised (within the last 90 days) and at risk of hospital readmission. Two participants were excluded from the study because interviewers noticed afterwards that they did not meet the polypharmacy inclusion criteria (≥5 daily prescribed medicines). Each OA was asked to name their most significant IC if they had one. ICs (any family member, neighbour or friend assisting a dependent OA with certain activities in their daily life) were invited to participate if they were involved in the OAs’ medication management. Being involved in medication management implies at least one of the following activities: accompaniment to health consultations, assistance in obtaining medication, the provision of medication, assistance in providing and/or taking medication, monitoring effectiveness and side effects, assistance in keeping medication schedules and organising home-care support [37,43].

An HP was also recruited for each participant. Professional caregivers are those employed to provide professional community health care services (i.e., nurses, nursing assistants, general practitioners, pharmacists). They were included in the study if the recruited OA identified them as the professional most involved in their medication management. Table 1 presents the specific inclusion/exclusion criteria for each category of participant. Research nurses partnering the project, from either the hospital centre or the community healthcare centre, identified older people who fulfilled the inclusion criteria and briefly explained the study to them. Potential participants were asked for permission for their name and telephone number to be given to the researchers. FP and MB then contacted OAs by telephone during the week following their hospital discharge and asked if they were interested in taking part in the study. In case of agreement, a first meeting was organised at the OA’s home in the next few days.

### 2.3. Data Collection

**Figure 1:**
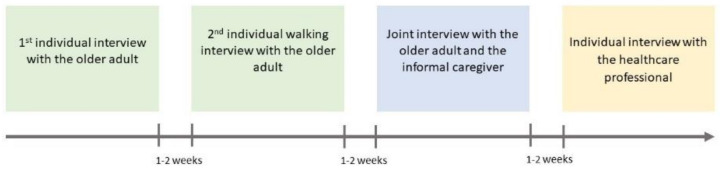
Data collection phases.

**Figure 2:**
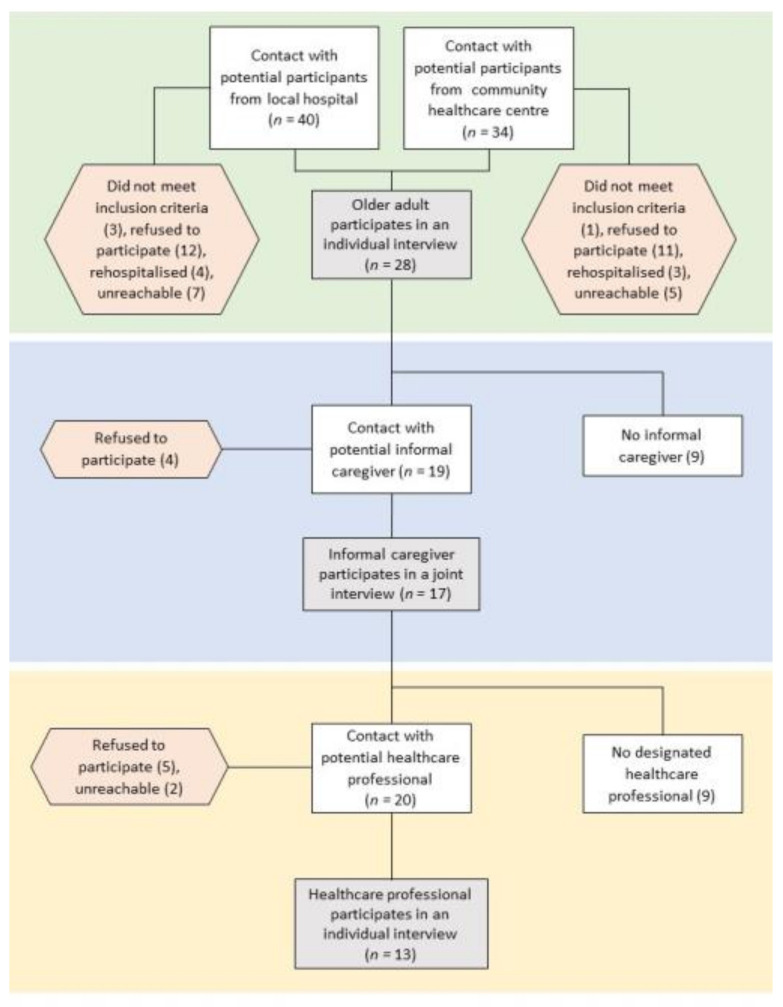
Description of the enrolment procedures.

FP and MB were responsible for data collection, and both have significant experience in qualitative methods. They prepared themselves by training together before the first interview to ensure the methodological rigour of their data collection process. All interviews were audio-recorded and lasted between 30 and 90 min, depending on the elements that participants shared. At the end of each interview, FP and MB made notes on how it had proceeded. Each home-dwelling OA was supposed to participate in a total of three interviews (Figure 1). Enrolment procedures are described in Figure 2. FP or MB first contacted OAs by telephone, asking if they wanted to be involved in the study. If they did, FP or MB met them in their own homes to explain the study’s details, check whether they met the inclusion criteria and collect their signed written informed consent forms if they did. The first interview’s objective was to explore OA’s experiences, perceptions and lived experiences of their hospitalisation and return home, paying special attention to the information they had received about their treatment and its possible modifications, and the involvement of their ICs and HPs throughout the process. Participants were also asked to keep a week-long daily medication diary (Supplementary File S1). The second interview occurred 2 to 3 weeks later. The OA commented on and explained their completed medication diary. We used a “walking interview” model [44] to collect spatial information and situated data to complete the medication diary. The emphasis was placed on medication management at home and the ICs and HPs involved in medication practices. The initial data collection plan had to be adjusted in some situations: depending on the OA’s level of tiredness, FP or MB would decide to conduct two interviews, according to each one’s interview guide, at the first meeting. When an OA had forgotten to complete their medication diary, FP or MB completed it with them during the second encounter by asking, for example, “How does a normal day begin for you?”.

A third, joint interview with the OA and their IC (if available and pertinent) was organised at the OA’s home 1 to 2 weeks after the second interview. The meeting was organised by telephone, with the IC being asked if they wanted to be involved in the study. If the IC had already been present during one of the two previous interviews, then the third interview guide had been used at that time. At the beginning of the meeting, FP or MB explained the study’s details, checked whether ICs met the inclusion criteria and collected their signed written informed consent forms if they did. The third interview’s goal was to explore the interactions between OAs and their ICs with particular regard to medication management. The hypothesis was that the primary IC would be deeply involved in the OA’s experience of medication management, and that the caregiver’s beliefs and perceptions about this might be similar to, overlap with or be different from the OA’s.

Finally, a semi-structured interview was conducted with the designated HP (if available and pertinent) in their workplace or by telephone. The meeting was organised by telephone, with the HP being asked if they wanted to be involved in the study. Again, at the beginning of the meeting, FP or MB explained the study’s details, checked whether they met the inclusion criteria and collected their signed written informed consent forms if they did. This was to explore their viewpoints about interactions between OAs, ICs and HPs on medication management at home after hospitalisation. They were also asked about their patient’s attitude towards medication changes after hospitalisation and any potential difficulties they subsequently faced. In agreement with the project’s field partners and stakeholders, these interviews took place in HPs’ workplaces (community healthcare centre, medical practice office or pharmacy), during working hours, and 1 to 2 weeks after the third, joint interview.

Due to the global COVID-19 pandemic, data were collected at two different times: November 2019 to March 2020 and September 2020 to November 2020. Since the population involved in this study was particularly vulnerable to COVID-19, the research team quickly decided to interrupt data collection and start again as soon as this became possible.

Throughout the data collection process, the research team considered the effect that the interview setting might have on the interviewees’ discourse. So that our study was not associated with medication adherence monitoring, the interviewers insisted on participants’ anonymity and on the absolute confidentiality of the information shared. A non-judgmental attitude was systematically encouraged, and the interviewers repeatedly mentioned that their objective was understanding the interviewees’ lived experiences.

### 2.4. Data Analysis

The researchers decided that data saturation had been reached after interviewing 28 OAs, 17 ICs and 13 HPs. Due to the significant quantity of data collected, interviews were transcribed by a company specialised in this field (https://www.amkfrance.fr/, accessed on 18 August 2021). FP, MB, GM and FR read the interviews transcripts to check their reliability and accuracy, and then performed a thematic content analysis with the help and expert guidance of MDRC and HV. Based on an inductive approach, thematic content analysis [45] allowed us to identify the themes emerging from the data and provided a rich, detailed account of the data set. Once the interviews had all been transcribed, the 60 interviews were distributed among MB, FP, GM and FR. The first step required becoming familiar with the data by reading and annotating the transcriptions. The coding process proved challenging because it occurred during Switzerland’s COVID-19 quarantine period. Each investigator coded the interviews allotted to them and, in parallel to this, regular video meetings were held to agree on the shapes of the thematic trees each of them was creating and on how to label the different categories and themes on those trees. Themes were compared by the different members of the analysis team until a consensus had been reached. Once a single thematic tree had been agreed upon and to lighten the data file, a second thematic tree was developed using only the most significant verbatim quotations by the participants in order to make synthesis easier.

## 3. Results

The socio-demographic characteristics of the three types of participants are presented in Table 2. On average, elderly participants had 13 ICD-10 diagnoses in hospital (range 4 to 21) and an average of nine prescribed medications at hospital discharge (range 5 to 21). Seventeen ICs designated by our 28 OAs participated in the study, the difference between the numbers being mainly because not all the OAs had ICs. Even if they did, ICs were not always involved in medication management, and they did not always agree to participate.

Our thematic content analysis allowed us to highlight three major themes illustrating the different types of beliefs about how medication management affects polymedicated home-dwelling OAs and their ICs. The HPs also added their viewpoints on the beliefs held by OAs and ICs. The first major theme explores the beliefs and preferences of OAs and ICs regarding the information transmitted by HPs about their medical treatments. The second major theme examines beliefs directly associated with the prescribed polypharmacy and which are revealed through the ways OAs adhere to their medication treatment. Using beliefs as a starting point, the third major theme describes OAs’ and ICs’ daily lived experiences of polypharmacy, lying somewhere between believing in being a proactive, self-reliant patient and the acceptance of submitting to and following physicians’ prescriptions.

### 3.1. Stance Vis-à-Vis the Information Transmitted by Healthcare Professionals—A Continuum between Accepting and Distancing Oneself from Physicians’ Prescriptions

OAs and their ICs acknowledge the information they receive from HPs about their medication prescription in a number of different ways. Initially, some OAs expressed their wishes not to be particularly involved in or informed about their polypharmacy. They affirmed that they were simply not interested in knowing the precise reasons why they were receiving different medication treatments. Some even expressed their refusal to get too involved in case they “found out something that I didn’t want to know” (OA25, 2nd interview). Some OAs justified their disinterest by asserting their trust in the HPs looking after their prescriptions: “They [prescribing doctors] know more about medication than I do” (OA27, 1st interview). ICs also described how their trust in the HPs responsible for prescribing medication justified their lack of detailed personal interest. This stance was particularly visible among the ICs who were also OAs’ spouses. However, the trust shown in the prescribing HPs on the appropriateness of medication prescriptions was not always bestowed upon the professional caregivers who administered those medicines day-to-day. IC15 for example expresses her trust in her husband’s prescribing physician and was not interested in taking an active part in the decision-making process. Despite this, she displayed a more active and suspicious stance when it came to the day-to-day administration of medication by the community healthcare centre, as one home-care assistant described:

**HP15**

Interviewer: *(…) It’s coming back to me now, but I remember that Mrs [IC15] was telling me that she wanted to remove the drugs [from their packaging].*

HP15: *Yes, so that’s what we do at her place; we leave them in their blister packs and then she takes them out like that, and that allows her to monitor them too, and there you go.*

Regarding explanations about changes made to medication regimens—whether they were well or poorly communicated by HPs—we got the same answer from OAs. This went so far as one OA declaring that even if the reasons for medication changes had been explained to him, it would have been of no use because “I don’t know anything about medicines” (OA11, 1st interview). Furthermore, even when information was transmitted to OAs, they described their apparent passivity: “but then again, I didn’t listen because it’s of no use to me” (OA21, 1st interview).

A different stance involved actively seeking out information and seeing that communication as necessary and essential. This was reflected by some ICs and OAs who described how they either relied on the medication’s “usage instructions” (OA05, E1) or preferred them over the instructions from their prescribing physician: “Sometimes, maybe, I’ll ask the doctor, but usually I still look at them. (…) I like having a look at what I’m taking or else what they are giving me. I always take a look” (IC11, wife, 2nd interview). That willingness to take ownership of a polypharmacy treatment and any change to it was also expressed by OAs keeping all the information given to them about their treatment and any potential changes, notably with a view to improving the flow of information between different HPs. As OA13 explained: “Yes, if we go in there, yes, I’ll ask about it, and I’ll note down what it is for (OA13, 2nd interview). The ICs who are the children of OAs generally take this stance and welcome any and all information about the medication prescription that might be useful. One IC, for example, regretted the general lack of information passed on about the treatments prescribed for her father: “I don’t know. They might have given you an explanation when I wasn’t there. I get the usage instructions, the envelope and that’s it: new treatment, full stop. Okay, sometimes they say, well, we are increasing the dosage for the diuretic, or we’re decreasing it for this reason, but there you go” (IC12, daughter, 1st interview). One situation highlighted the mismatch that can exist between the perceptions of ICs and OAs on the subject of communication with HPs. One IC spoke about having been very satisfied with the information given to her by a physician at the hospital. However, her OA said, “No. They didn’t tell me. They brought them to me. In the morning, there was this. At lunchtime, there was another one. And in the evening, there were others. There you go. Yes, well, they didn’t tell me that I had to change, I don’t know why. I was in their hands” (OA01, 1st interview).

The acceptance of or withdrawal from the information communicated by HPs, together with OAs’ and ICs’ preferences and how they went about seeking information about polypharmacy were all linked to their very personal stances towards HPs and the bio-medical sector in general. There was also a link to beliefs about medication itself, as was underlined by the OA who did not wish to find out too much, in case he found out something he would rather not know about.

### 3.2. Day-to-Day Polypharmacy Management—The Effects of Beliefs and Lived Experiences

Our findings revealed nuanced beliefs about the effectiveness of the medications prescribed. In general, they were perceived to be both useful and effective, but certain specific medicines were not. For example, some OAs experienced analgesics as ineffective, which justified not taking them, whereas others did find them effective, justifying their strict adherence to their prescription. One professional home-caregiver explained an OA’s belief about the ineffectiveness of the analgesics prescribed to her, which was reflected in her daily medication management: “So, anyway, she doesn’t find Dafalgan [paracetamol] very effective, so she doesn’t take it. Even though we tell her that in association with other analgesics it’s good to take it. Well… it’s her choice, after all” (HP04). Another professional home-caregiver said, “So, sometimes it was just forgotten, sometimes it was because she thought that it did her more harm than good. So they were the main reasons. Then, she was a bit reticent about the analgesics” (HP07). On the contrary, one OA explained that “If it leaves me feeling good [Dafalgan], I usually keep taking it! (Laughs)” (OA05, E2).

OAs and ICs expressed their worries about the risks of becoming dependent on some medication. Concerning sleeping pills, one IC stated, “They might… If you start taking them, then you have to take them all the time” (IC27, wife, 2nd interview). These words were similar to those of one OA who refused to take the sleeping pills prescribed for him at the hospital: “And I said, ‘I’m not taking that!’ I’ve never taken sleeping pills before. I’m not starting now!” (OA05, 2nd interview). There were real divergences in beliefs between OAs and ICs, as witnessed during this exchange about managing analgesics:

**OA07 and IC07, daughter, interview 1**

IC07: *As to pain, she waits too long.*

Interviewer: *She waits too long?*

OA07: *Yes… listen. If I take it all, in the end, it doesn’t work anymore when it really does hurt.*

Interviewer: *Right. So, you feel more relief like that, yes?*

OA07: *But when I’m not in pain, I don’t take it. (…) You shouldn’t take drugs when you don’t need them.*

The parallel between becoming dependent on medication and addicted to drugs was mentioned several times. The belief that medication necessarily implied some negative effects, such as stomach ache, were at the origin of a number of practices carried out when taking medicines. According to one IC, taking medication at mealtimes prevented certain gastric pains because OAs have “something in their [stomach]” (IC15, 2nd interview). One OA shared a tip about taking medication with some of the fermented drink, kefir: “So, I take every medicine with some of that. Why? Because it creates a kind of a lining in the [stomach]. It regulates the intestinal flora. It’s really great” (OA22, 1st interview).

OAs had strong opinions about the number of medications prescribed to them, declaring, for example, that “You shouldn’t overdo it on the drugs!” (OA05, 2nd interview). The desire to limit the number of medications consumed daily also reflected the potential dangers they can represent, especially in terms of drug–drug interactions. On that issue, one OA advised: “But you have to be careful what else you take on the side.” (OA10, 2nd interview). One HP, a general practitioner, also explained that the length of a medication prescription can make some patients unhappy. She explained how she dealt with such complaints.

**HP28**

HP28: *(…) So, we always try to remove what isn’t absolutely necessary, that’s for sure. The less he takes, the better he is, in general.*

Interviewer: *So that’s something you discuss with your patients?*

HP28: *Yes, yes, yes, yes. Then there are those who really cling on to their regular medication, you know? They’re the opposite. I try to remove a drug, and they absolutely don’t want to—for their stomach or things like that. There are some like that. Each person is different. But when it comes down to it, we try to shorten the length of medication prescriptions.*

Several OAs and ICs shared beliefs about prescribed polypharmacy, in terms of its effectiveness and utility, but also about the potentially negative effects of medicines. Beliefs about medicines are an integral factor in how patients adhere to their polymedicated prescriptions and are usually based on individuals’ personal daily lived experiences.

### 3.3. Turning the Obligation to Manage Medication into an Automatism: Everyday Resourcefulness

The majority of OA–IC dyads or OAs on their own mentioned the normalcy or automatism linked to taking medication every day. When the investigators asked about the tricks put in place to enhance or encourage good treatment adhesion, many OAs and ICs simply said that it was a habit:

**OA02 and IC02, wife, 1st interview**

Interviewer: *What’s your trick for not forgetting them?*

OA02: *Nothing, it’s just habit.*

Interviewer: *It’s a habit.*

IC02: *(Laughs) You’re just so used to it, I think. I don’t know.*

The term ‘automatism’ was used by numerous OAs and ICs, sometimes in relation to how they functioned as individuals: “I work like that (…). If I’ve got to do something, I know that I’ve got to do it” (OA21, 1st interview). OA’s individual functioning also refers to the professional activity carried out during their life: one OA explains that the way he takes medicines automatically and unquestioningly is linked to his professional career in the military where he had been used to following orders without question. Without mentioning them explicitly, OAs and ICs put in place tricks to help with good daily adhesion to treatment. These tricks become invisible because of their routine or automatic nature, but they are nevertheless there. Diverse inventive tactics for creating routine were revealed during the interviews, such as ICs putting pills in coloured box lids so that OAs would notice them and be able to identify them thanks to the contrasting colours. Also, weekly pill-boxes were opened in advance to facilitate the OA’s ability to access medication when necessary. Time markers were also put in place to remind OAs when to take their medication throughout the day. Most participants, for example, associated taking their medication with mealtimes so as not to forget them. ICs setting alarms on mobile telephones was also a frequently used technique, as were reminder notes slipped into OAs’ wallets or purses. The weekly pill-box was an almost unavoidable time marker as the days of the week are written on its different trays to avoid confusion.

The weekly pill-box was also used as a spatial reminder to take medication because all the medicines were centralised, encouraging better organisation. As one IC explained, “That way, there, I can’t forget them. They are all there” (IC11, 2nd interview). OAs or ICs select other strategic locations to help medication management: a note stuck to the fridge can act as a reminder, evening or morning medication can be placed on the bedside table, or preparing tomorrow morning’s medication can be done on the kitchen table the evening before. Thus, OAs cannot help but see their reminders—they are “obliged to see them” (OA01, 1st interview)—and the risk of forgetting their medication is eliminated.

The act of taking several medicines daily is also seen as an obligation for OAs, without that being in contradiction with the habit or automatism that characterises that act. The discussions around maintaining a medication diary—and more precisely around the question of “Are you happy taking this medicine?”—precipitated some strong reactions, as illustrated by how one OA filled out his diary (Supplementary File S2). He commented: “Asking me whether I’m happy or not taking a medicine is like asking me whether I’m happy getting an injection before they give you an injection. (Laughs) (…) There you go—it’s an obligation” (OA18, 2nd interview).

The presence of physical symptoms or fear of the consequences of not following their polypharmacy regimens are the sources of patients’ feelings of an obligation imposed on them by their underlying illnesses: “Because I think that one or two days won’t hurt me, but after that I’ll start to get pains” (OA08, 2nd interview). A level of tension, characterised by frustration or a feeling of resignation, may then emerge, and this experience is lived differently by OAs and ICs:

**OA11 and IC11, wife, 2nd interview**

OA11: *Bah! I take it because I’m obliged to, otherwise you think I’m taking…*

Interviewer: *Because you have to do it.*

IC11: *But you are satisfied [with your treatment] at the moment. If you don’t take them, you…*

OA11: *But if I want to feel as good as I do now, I’m obliged to take that rubbish.*

For this IC, that obligation was internalised and her husband was “satisfied”, but nevertheless his feelings revealed a greater level of internal conflict. One HP, a home-care assistant, shared the sentiment that her patient was “fed up with swallowing all these tablets” (HP04). To avoid this conflict, some medications, perceived by OAs as being non-essential to the body’s well-being, were simply not taken. One OA revealed his way of doing things transparently by trying to accommodate his own values and the medication prescription imposed upon him: although the community healthcare centre prepared his weekly pill-box for him, he administered his daily medication independently and frequently decided not to take them all. When the home-care assistant noticed that some pills were still inside the pill-box, he would say to them: “Yes, so I say, ‘I didn’t feel the need to take them’, and then it’s okay. There” (OA28, 2nd interview). Other lived experiences were also shared, such as by an OA who declared: “If I don’t have my medication, I panic, yeah?” (OA2, 2nd interview).

The monitoring exerted by HPs, perceived by OAs as an injunction to take care of themselves, can become an external source of feeling an obligation: “I know that—goodness—you have to take care of yourself, whether you want to or not. (Laughs)” (OA14, 1st interview). The presence of medicine cabinets in OAs’ homes, locked by home-care assistants so as to avoid patients ‘helping themselves’, symbolises the obligation not to self-medicate. One HP, a home-care assistant, explained:

**HP20**

HP20: *So, that’s to say it’s to avoid, err, errors or the temptation of taking more. For example, because of his dependence on benzodiazepines—Xanax, for example—it would be annoying if he… (…) went to get something more, some sleeping pills or…*

Interviewer: *Yeah, yeah, of course. It’s actually a safety measure.*

HP20: *Yes, although I’ve never felt very comfortable setting up this type of thing. (…) I find that it’s a little bit of a heavy-handed method, but there you go.*

The management of analgesic medication also illustrates the accepting stance taken by some OAs: despite their perceived lack of effect, OAs continued to take them because, “No, but they [hospital staff] said, you have to take that to regulate it [the pain]” (OA04, 1st interview). Some medications, contrarily to analgesics, are not designed to relieve short-term physical symptoms but are instead preventive, such as anticoagulant medication or cholesterol medication. One IC, who was actively involved in preparing and administering her father’s medication, explained: “It’s not as if you say, ‘Yay! I took my Sintrom.’ That would be wrong. You take it because it’s your duty.” (IC17b, daughter, 2nd interview).

The feeling of resignation emerged from the tension between using intrinsic beliefs as a definition of one’s own identity and “submitting” to HPs’ recommendations. Several OAs defined themselves as “not being on medication” (OA10, 1st interview) and “anti-medicines” (OA22, 1st interview), or they stipulated that “I’m not really into medication unless I’m obliged to be” (OA09, 1st interview). One HP, a home-care assistant, explained that one particular OA was, “(…) in general, reticent about taking her medication (…). So, according to her, the fewer medicines she takes, the better she is” (HP07). One IC pointed out her feelings of resignation, that she felt resulted from the tension between her own intrinsic values and a form of “submission” to what was expected of her as a loyal IC: “I’m against medicines, but then I have to go with it” (IC15, 1st interview).

Lastly, a feeling of indifference and disconnection from the daily consumption of medicines also emerged, as with this OA:

**OA25 and IC25, wife, 2nd interview**

OA25: *Absolutely nothing at all. You could make me take 50; I’d take them and that would be fine, but I don’t worry about knowing whether it’s going to do me any good or not.*

Interviewer: *Okay. So, it’s something that you observe from afar, and…*

IC25: *Do you know what I called my husband when he was young? I still call him that—my carefree beau. He doesn’t care about anything. He’s lucky.*

OA25: *I am totally indifferent about it.*

This apparent disconnect nevertheless relies on a form of negotiation or contract among some OAs: “(…) So long as I don’t have diarrhoea and I’m not vomiting, then I’m not going to moan” (OA29, 1st interview). In the absence of any side effects, many OAs explained that they took their medication “without grumbling” and that situation seemed to suit them.

The meaning attached to the act of having to follow one’s medication prescription was one of normalcy and automatism, but also sometimes a sense of obligation or imposition. Systems of negotiation had been put in place, mainly by OAs, to accommodate their beliefs and feelings of obligation: defining themselves as “anti-medicines” yet taking all or most of their pills every day seemed to be evidence of the internal dynamics and rationales for action that underpinned their daily adhesion to polypharmacy treatments. Table 3 summarises the main interview findings. Polypharmacy highlighted some of the power and relational issues existing in OAs’ and ICs’ dealings with HPs when they were given or searched for information on that polypharmacy. The information transmitted by HPs came up against a number of beliefs and reactions, somewhere between submission and passivity and even questioning the very need for medicines. This may help to explain a tendency for OAs and their ICs to sometimes not want to know too much about or even get involved in their polypharmacy treatments. Even when a treatment was perceived to be useful and effective overall, beliefs about the potentially negative or dangerous side effects of some medicines emerged and became mixed up in OAs’ and ICs’ daily lived experiences. Thus, the meaning given to taking several medicines daily was not only habit; feelings of obligation emerged, revealing inventive systems of negotiation with oneself into which OAs tried to integrate their own beliefs.

## 4. Discussion

The present study aimed to identify and analyse the beliefs of polymedicated home-dwelling OAs and the ICs assisting them with that polypharmacy by asking them about their daily medication practices. The OAs interviewed estimated that their prescribed medications were useful and effective overall, and most of them did not mention having unilaterally decided to stop taking them or reduce dosages. This was in line with other studies that established a link between perceived effectiveness and daily use [46]. Nevertheless, OAs entertained other beliefs, notably about analgesics. This is despite the fact that analgesics should have a noticeable, fairly rapid effect on the body (even if only in the short-term), something which is not true of other types of medication. Some OAs underlined that the non-effectiveness of analgesics led them not to take them [47]. Other authors, however, have noted different results, describing how analgesics prescriptions were adhered to assiduously [13], differently to other medication whose long-term effects were directly perceptible [48]. OAs notably shared specific beliefs about the risks of becoming dependent on analgesics or sleeping pills. The belief that medication has negative effects is revealed in the literature [16,17]. Our interviews revealed strong identity-based stances as several OAs and one IC defined themselves as “anti-medicines” or felt a sense of resignation or obligation to adhere to their medication prescription, and this was also visible in the literature [16,20].

Giving OAs and their ICs the opportunity to share their beliefs about and attitudes to polypharmacy with HPs, whether home-care assistants, pharmacists or general practitioners, could become an essential stage in working towards better treatment adhesion. This could be particularly so when the length of prescriptions starts to become a significant worry in itself. One study underlined that OAs are generally less aware of the indications for each medication than other populations [26]. The length of a medication prescription leads to specific mechanisms of action, as one general practitioner mentioned: her objective was to aim for the shorter prescriptions that his patients preferred. Indeed, polymedicated OAs are generally in favour of this process [27]. Although it has been reported that a doctor-centred communication style is likely preferred by older patients and patients with a lower level of education [49], shared-decision making and patient priority-directed care for OAs with multiple chronic conditions and their ICs is promoted [50,51]. ICs, especially OAs’ children, seem to be particularly inclined to take an active role in care decisions, as do some OAs. OAs who do not wish to get involved in care decisions express their feelings of a lack of legitimacy in the face of HPs, and this is another belief that will have to be deconstructed among older populations. Making the HP–patient relationship more symmetrical or balanced will require giving some power back to the patient, but empowered patients are more motivated to adhere to their treatments [52].

Temporal and spatial reminders—whether these involve alarms, memo boards, spreading medication around homes in strategic locations or taking medication at meals or coffee breaks—are a means of incorporating medication into OAs’ daily routines or that of their dyad with their ICs. The emphasis on medication routines has been noted in situations of complex medication regimens [53]. The integration of medication intake into daily routines and activities is especially observable among polymedicated OAs because they are retired and more likely to be sedentary [54], and other researchers have concluded similarly [12,13,14]. Nevertheless, the question, “What are your strategies or tricks for ensuring that you take your medication properly?” usually resulted in a shrug and a reply that described how normal and automatic these acts were. At first glance, the *invisibility* of these strategies brings to mind the notion of “extraordinary normalcy”: “consistent and familiar domestic routines can provide a sense of normalcy in the home and a means of managing relational shifts that occur when someone requires additional care” [55] (p. 74). Understanding OAs’ routine medication practices inside their own homes—a mirror of their level of appropriation of their own medication treatments—is essential to target recommendations to reinforce medication adherence among this population. Indeed, the current evolving demographic and epidemiological challenges of an ageing population imply longer, more active lives, but lives frequently accompanied by chronic illnesses and thus multiple complex medication treatments [2,3]. In Switzerland and elsewhere, public health systems support and favour OAs living at home, as do OAs themselves [56,57]: the expansion of *ageing in place* approaches will become ever more essential as home settings evolve into care settings [15,58].

The present study also showed the importance of examining daily routines and practices in order to better understand the medication beliefs of OAs and ICs. Their daily medication practices and strategies were also major negotiating tools with which to help match their values and beliefs to medication prescriptions that should be followed conscientiously. By asking, “Tell me about a typical day. What is the first thing you do in the morning?” we revealed OAs’ meaningful daily habits and practices and brought to light a form of experiential knowledge. According to Cromby [39], the definition of the term ‘*belief*’, which is rooted in discourse psychology, enables us to think of this experiential knowledge as part of a system of beliefs. Indeed, the strategies put in place to encourage medication adherence in line with prescriptions are built on OAs’ daily lived experiences of those medication prescriptions and on beliefs whose origins are multiple and the reflection of co-construction: “Since thinking is already a social process, treating belief solely as a psychological state may obscure how it is already embedded in ‘meaning, experience, emotion, order, individuality, thought, action, identity, sociality, rationality, symbolism and power’ [59] (p. 10)” [39] (p. 945). The belief system and rationales for action that daily lived experiences are the source of are complex and have multiple origins: one OA’s consumption of the fermented kefir drink, for example, may have originated from faith in a discourse far removed from Western biomedical references, from the physical sensation the drink procured, from the previous negative sensations it blocked out, or perhaps even something else. Considering the perceptions of multiple stakeholders in an exploration of OAs’ beliefs is a means of better grasping their nature and how they affect the daily management of medication at home. The particular methodology that we put in place—including individual interviews with OAs and joint interviews with OAs and ICs—enabled us to examine all their perceptions. The interactions and dialogue in the joint interviews provided a bonus of rich detail in comparison to studies using two individual interviews and then attempting to triangulate information. These elements supported previous work [60], highlighting the fact that joint interviews enabled researchers not only to collect all their participants’ different viewpoints but also to understand their perceptions of the dynamics and interactions at play within their own family.

## 5. Study Strengths and Limitations

The present study was able to analyse beliefs about polypharmacy by using an original qualitative methodology to collect the viewpoints of multiple stakeholders. This approach does not aim to triangulate information to identify treatment adherence among OAs, rather it seeks a broader perspective of their beliefs about polypharmacy. The study’s multidisciplinary dimension was a significant strength because health psychology and the nursing sciences were not only used in its construction but also in the analysis of its results.

The present article nevertheless has some limitations. The protocol involved the plan to systematically recruit one HP for each OA interviewed. However, this proved impossible for reasons of unavailability. For some HPs, mainly general practitioners, our research objective was not a priority, although each OA designated the main HP involved in their polypharmacy management. Working in the context of a pandemic compromised participant recruitment due to the vulnerability of our population of interest. COVID-19 and social distancing recommendations also compromised scheduled home visits, and two HPs and one OA had to be interviewed by telephone. Despite the limitations caused by COVID-19, we were still able to analyse the beliefs of home-dwelling OAs and their ICs with regards to polypharmacy, and our research goal was thus attained.

## 6. Recommendations for Practice and Research

Using joint interviews with OAs and their ICs helped us to collect multiple beliefs about polymedicated OAs and to concurrently hear elements of the discourse and interaction occurring between these stakeholders. The present article does not discuss these interactions and they will be the subject of broader methodological reflections in a future publication. One initial recommendation for researchers interested in polypharmacy among home-dwelling OAs would be to systematically consider their ICs’ perceptions of this topic because they fulfil a central role in day-to-day polypharmacy management. The perceptions of the HPs designated by the OAs are also precious sources of information, especially in the face of the latter’s beliefs.

Our results highlight the importance of HPs’ communication skills, which was reflected in the information retained by OAs and their ICs. Indeed, repeating or transmitting information about medication is not enough. HPs should pay particular attention to the information that OAs and their ICs perceive they were given and what they actually understood; they should listen to OAs’ underlying, basic beliefs about healthcare, medication and prescriptions; they should adapt their discourse to fit into the thought framework of OAs’ daily lives; and they should move closer towards a stance that encourages more shared decision-making. Carefully considering OAs’ daily medication practices and the knowledge they have accumulated through lived experiences can flatten the patient-perceived hierarchy between OAs, their ICs and HPs. Indeed, the strategies and tricks revealed by our methodology gave insights into underlying beliefs about medications. The notion of beliefs about medicines is regularly used in the literature to help explain or encourage behaviours relating to treatment adhesion during chronic illnesses such as diabetes, asthma or rheumatoid arthritis [61,62,63]. Therapeutic education programmes for patients, which often target a specific chronic disease, could emerge for polymedicated OAs, helping them to better comprehend the rationales for action underpinning the use of multiple medicines and encouraging better treatment adhesion. Current demographic trends will force us to pay greater attention to polypharmacy among home-dwelling OAs and how their practices and beliefs about polypharmacy affect their lives.

## 7. Conclusions

This study questioned the negative beliefs and preferences of home-dwelling older adults and their informal caregivers and healthcare professionals regarding their prescribed polypharmacy, and the meanings they attributed to this daily medication intake emerged. Underneath the seemingly routine and automatic act of taking one’s medication, older adults expressed their feelings of obligation or resignation towards polypharmacy. Any study of adherence to treatment, its issues and its determinants requires considering the points of view of multiple stakeholders. Beliefs about medication are directly linked to adherence and are based on patient’s own experiences, which is why contextualised everyday medication practices should be questioned and observed, both in research and in home care settings.

## Figures and Tables

**Table 1:** Participant inclusion and exclusion criteria.

Participants	Inclusion Criteria	Exclusion Criteria
Older adult	- Aged 65 or above - Man or woman - Recently hospitalised (in last 3 months) - Managing at least 5 different medications - Suffering from at least 2 chronic conditions - Living alone or in a couple, in a rural or urban area - With or without support from a community healthcare centre	Unable to speak and understand French
Informal caregiver	- Aged 18 or above - Designated by the older adult as the most significant informal caregiver involved in medication management	Unable to speak and understand French
Healthcare professional	Designated by the older adult as having a key role in medication management	- Student - Apprentice - Unable to speak and understand French

**Table 2:** Participants’ socio-demographic and professional characteristics.

Socio-Demographic and Professional Characteristics	Older Adults (*n* = 28)	Informal Caregivers (*n* = 17)	Healthcare Professionals (*n* = 13)
Sex			
Female	11	15	10
Male	17	2	3
Age			
Mean [range]	81.1 [66–94]	67.6 [48–86]	43.8 [28–58]
Relationship with the older adult			
Spouse/partner (%)		10 (58.8%)	
Children (%)		6 (35.3%)	
Daughter-in-law (%)		1 (5.9%)	
Work status or profession			
Retired (%)	28 (100%)	9 (52.9%)	
Employed (%)	0	7 (41.2%)	
Unemployed (%)	0	1 (5.9%)	
Nurse (%)			5 (38.5%)
Pharmacist/assistant pharmacist			4 (30.8%)
General practitioner/specialist (%)			4 (30.8%)
Number of medicines			
Mean [range]	9.0 [5–21]		

**Table 3:** Summary of the main interview findings.

Themes	Description
*Stance vis-à-vis the information transmitted by healthcare professionals—a continuum between accepting and distancing oneself from physicians’ prescriptions*	- Older adults (and a few informal caregivers) mentioned **having no interest in being involved or informed** - Informal caregivers (and a few older adults) expressed **their willingness to take ownership** of the polypharmacy treatment by seeking out information and asking questions.
*Day-to-day polypharmacy management—the effects of beliefs and lived experiences*	The way older adults and informal caregivers adhered to the medication prescription depended on their beliefs, based on their individual, personal daily lived experiences about: - **Non-effectiveness of medication** - **Risks of medication dependence** - **Negative effects of medication** - **Medication overuse**
*Turning the obligation to manage medication into an automatism: everyday resourcefulness*	Taking polypharmacy was experienced as: - A **habit** and an **automatism**: without mentioning them explicitly, older adults and informal caregivers put in place tricks and strategies to help them maintain good daily adhesion to treatment. - An **obligation**: underpinned by the fear of physical symptoms and the monitoring done by health professionals, both older adults and informal caregivers felt this. - Feelings of **frustration** and **resignation**: these were often described (mainly by older adults but also by some informal caregivers); negotiation systems had been put in place, mainly by OAs, to accommodate their beliefs (and their own definition as “anti-medicine”) and feelings of obligation.

## Data Availability

The data presented in this study are available on request from the corresponding author. The data are not publicly available due to ethical issues regarding qualitative methods.

## Supplementary Materials

The following are available online at https://www.mdpi.com/2227-9032/9/9/1204/s1: Supplementary File S1. The week-long daily medication diary; Supplementary File S2. An extract from the medication diary of OA18; Supplementary File S3. The completed 32-item COREQ (COnsolidated criteria for REporting Qualitative research) Checklist.
