# Supplementary material for: Beliefs about Polypharmacy among Home-Dwelling Older Adults Living with Multiple Chronic Conditions, Informal Caregivers and Healthcare Professionals: A Qualitative Study
Source: Healthcare (Basel). 2021 Sep 13;9(9):1204. doi: 10.3390/healthcare9091204 (PMC8466336; doi:10.3390/healthcare9091204)
Supplement: Supplementary file 1 [file healthcare-09-01204-s001.zip › Supplementary file 1.pdf]

Journal de vos pratiques de médication

Gestion des médicaments chez la personne vivant à domicile avec des conditions médicales chroniques

Jour : ………………………………………………… Date : …………………… Code du participant : ………………

Cher participant, voici quelques questions auxquelles nous vous invitons à répondre lors de vos prises de médicaments :

**Moment de la journée de prises de médicaments**	**Quels médicaments avez-vous pris ?**	**A quel endroit et à quel moment avez-vous pris vos médicaments ?**	**Qui était présent lors de votre prise de médicaments ?**	**Étiez-vous plutôt content de prendre vos médicaments ? Ou plutôt mécontent ?**	**Avez-vous d'autres commentaires ou sentiments à nous transmettre à propos de vos prises de médicaments ?**
**Matin**					
**Midi**					
**Après - midi**					
**Soir**					
**Nuit**					

Basé sur les articles scientifiques suivants :

Dew, K., Norris, P., Gabe, J., Chamberlain, K., & Hodgetts, D. (2015). Moral discourses and pharmaceuticalised governance in households. *Social Science & Medicine, 131*, 272-279

Knight, D. A., Thompson, D., Mathie, E., & Dickinson, A. (2013). 'Seamless care? Just a list would have helped!' Older people and their carer's experiences of support with medication on discharge home from hospital. *Health Expectations, 16*(3), 277-291

Consignes :

- Chaque jour, pendant une semaine, vous pourrez compléter ce document aux moments où vous prendrez vos médicaments ;
- Vous pouvez indiquer les médicaments par leur nom, marque, forme/couleur, ou de tout autre façon qui fait sens pour vous ;
- Ce document sera consulté seulement par les membres de l'équipe de recherche ;
- Il est important que vous notiez tous les médicaments que vous avez pris, ceux prescrits par votre médecin, à l'hôpital, mais aussi les autres médicaments que vous êtes amené à prendre ;
- Vous pouvez demander de l'aide à un proche ou à un soignant pour remplir ce document ;
- Ce document n'a pas pour but de contrôler vos pratiques mais il sera un support pour notre discussion lors du deuxième entretien.

Vous pouvez contacter les membres de l'équipe de recherche en cas de question :

Responsable du projet :

Prof. Henk Verloo
Haute École de Santé, HES-SO Valais – Wallis
Chemin de l'Agasse 5, 1950 Sion
henk.verloo@hevs.ch
Tel. +41 27 606 84 24

Collaborateurs :

Prof. HES Ass. Filipa Pereira
Haute École de Santé, HES-SO Valais – Wallis
Chemin de l'Agasse 5, 1950 Sion
filipa.pereira@hevs.ch
Tel. +41 27 606 84 42

Dr. Maria del Rio Carral
Université de Lausanne
Quartier UNIL-Mouline - Bâtiment Géopolis - CH – 1015 Lausanne
maria.delriocarral@unil.ch
Tel. +41 21 692 32 56
