# Supplementary material for: Beliefs about Polypharmacy among Home-Dwelling Older Adults Living with Multiple Chronic Conditions, Informal Caregivers and Healthcare Professionals: A Qualitative Study
Source: Healthcare (Basel). 2021 Sep 13;9(9):1204. doi: 10.3390/healthcare9091204 (PMC8466336; doi:10.3390/healthcare9091204)
Supplement: Supplementary file 1 [file healthcare-09-01204-s001.zip › Supplementary file 2.pdf]

74 PNR Système de santé
Programme national de recherche

Hes·so VALAIS WALLIS Haute Ecole de Santé Hochschule für Gesundheit

Unil UNIL | Université de Lausanne

CHUV

Hôpital du Valais Spital Wallis

cms Région de Sion

Journal de vos pratiques de médication

Gestion des médicaments chez la personne vivant à domicile avec des conditions médicales chroniques

Jour : .... Date : .... Code du participant : PAg-18

Cher participant, voici quelques questions auxquelles nous vous invitons à répondre lors de vos prises de médicaments :

Moment de la journée de prises de médicaments	Quels médicaments avez-vous pris ?	A quel endroit et à quel moment avez-vous pris vos médicaments ?	Qui était présent lors de votre prise de médicaments ?	Étiez-vous plutôt content de prendre vos médicaments ? Ou plutôt mécontent ?	Avez-vous d'autres commentaires ou sentiments à nous transmettre à propos de vos prises de médicaments ?
**Matin**	– Ass Cardio-Mepha 100mg<br>– Meto Zerok 50<br>– Epril submite 5mg	- Après le petit-déjeuner<br>- A la salle à manger	Mon épouse	Par obligation !	Non
**Midi**					
**Après - midi**					
**Soir**	-Sintrom 1 mitis<br>-Atorvastatin 80mg	A la cuisine avant d'aller dormir	Mon épouse	Par obligation !	Non
**Nuit**					

Basé sur les articles scientifiques suivants :
Dew, K., Norris, P., Gabe, J., Chamberlain, K., & Hodgetts, D. (2015). Moral discourses and pharmaceuticalised governance in households. *Social Science & Medicine, 131*, 272-279
Knight, D. A., Thompson, D., Mathie, E., & Dickinson, A. (2013). 'Seamless care? Just a list would have helped!' Older people and their carer's experiences of support with medication on discharge home from hospital. *Health Expectations, 16*(3), 277-291
